# Can chronic spontaneous urticarial produce symptoms of neuropathic pain?^⋆^

**DOI:** 10.1016/j.abd.2022.06.004

**Published:** 2023-02-20

**Authors:** Gülhan Gürel, Hikmet Saçmacı

**Affiliations:** a Department of Dermatology, Faculty of Medicine, Afyonkarahisar Health Sciences University, Afyonkarahisar, Turkey; b Department of Neurology, Faculty of Medicine, Bozok University, Yozgat, Turkey

**Keywords:** Chronic spontaneous urticarial, Neuropathic pain, Pruritus

## Abstract

**Background:**

Chronic spontaneous urticaria (CSU) is a condition that is associated with recurrent pruritic hives and/or angioedema lasting for more than 6 weeks and is known to affect 1% of the population. Neuropathic pain can be defined as abnormal pain in the peripheral or central nervous system following injury and results from dysfunctions in the peripheral or central nervous system without peripheral nociceptor stimulation. Histamine appears in the pathogenesis of both the CSU and diseases of the neuropathic pain spectrum.

**Objective:**

To evaluate the symptoms of neuropathic pain in patients with CSU using scales.

**Method:**

Fifty-one patients with CSU and 47 sex- and age-matched healthy controls were included in the study.

**Results:**

The results of the short-form McGill Pain Questionnaire revealed the scores in the sensory and affective domains, Visual Analogue Scale (VAS) scores and pain indices to be significantly higher in the patient group (p < 0.05 for all cases), while the overall pain assessment and sensory assessment based on the Self-Administered Leeds Assessment of Neuropathic Symptoms and Signs (S-LANSS) pain scale were also significantly higher in the patient group. Based on the assumption that scores of > 12 indicated neuropathy, 27 (53%) of the patients in the patient group and 8 (17%) in the control group were found to have neuropathy (p < 0.05).

**Study limitations:**

Cross-sectional study, small patient sample and use of self-reported scales.

**Conclusion:**

In addition to itching, patients with CSU should be aware of the potential for the association of neuropathic pain. In this chronic disease that is known to affect the quality of life, using an integrated approach with the patients and identifying accompanying problems are as important as treating the dermatological disorder.

## Introduction

Chronic spontaneous urticaria (CSU) is a condition that is associated with recurrent pruritic hives and/or angioedema lasting for more than 6 weeks, and is known to affect 1% of the population.^1^ Drugs, foods, food additives, infections (bacterial, viral, and fungal), parasitic infestations, allergens and malignancies have all been identified in the etiology of CSU, although the cause cannot be identified in 50% of cases.^2^, ^3^ Vasoactive mediators released from dermal mast cells play a key role in the pathogenesis of CSU. Despite the presence of eicosanoids, cytokines and proteases, histamine is the major cytokine affecting the H1 (85%) and H2 receptors (15%) in the skin. The binding of histamine to H1 receptors causes pruritus (stimulation of C fibers) while binding to the receptors in capillaries leads to vasodilation, increased vascular permeability and edema.^4^

Neuropathic pain can be defined as abnormal pain in the peripheral or central nervous system following injury and results from dysfunctions in the peripheral or central nervous system without peripheral nociceptor stimulation.^5^ Neuropathic pain is a common problem in clinical practice and can be challenging to diagnose due to methodological differences in definition and assessment, although its estimated prevalence is 7%–10% in the general population.^6^ The mechanisms of pain formation are highly diverse and have yet to be fully clarified. Recent studies have reported that mast cells accumulate around nerve sheaths and play a role in the pathophysiology of neuropathic pain by secreting a wide variety of cytokines, including histamine.^7^

Histamine appears in the pathogenesis of both the CSU and neuropathic pain spectra of disorders. The present study investigates the symptoms of neuropathic pain in patients with CSU using scales, as the simplest and easiest method.

## Method

### Study sample

This cross-sectional, questionnaire-based and case-controlled study was approved by the local ethics committee (Decree nº 2017-KAEK-189_2019.07.10_04) and was conducted in accordance with the principles of the Declaration of Helsinki.

After obtaining their informed consent, 51 patients with CSU and 47 sex- and age-matched healthy controls were included in the study. All of the participants were above the age of 18 years. Excluded from the study were those with pregnancy, lactation, alcohol consumption, presence of a major central nervous system disease, cognitive impairment and psychiatric comorbidity. Only CSU patients were included in the study. Patients with chronic inducible urticaria and acute urticaria were excluded.

The body weight and height measurements were obtained for the calculation of Body Mass Index (BMI), as well as sociodemographic data, disease duration, comorbid diseases, drug use, presence of angioedema and clinical examination results of patients with CSU presenting to dermatology outpatient clinics.

### Measurements

#### Urticaria Activity Score for 7 consecutive days (UAS7)

Is an established and widely accepted patient-reported outcome tool for the prospective measurement of CSU activity. UAS7 scores of ≤ 6 indicate well-controlled, 7–15 mild, 16–27 moderate and 28–42 severe urticarial.^8^

#### The short-form McGill Pain Questionnaire (SF-MPQ)

The main component of the SF-MPQ comprises 15 descriptors (11 sensory; 4 affective) that are rated on an intensity scale of 0 = none, 1 = mild, 2 = moderate or 3 = severe, and three pain scores are derived from the sum of the intensity rank values of the words chosen for sensory, affective and total descriptors. The SF-MPQ also includes the Present Pain Intensity index of the standard MPQ and a Visual Analogue Scale (VAS).^9^

#### The Self-Administered Leeds Assessment of Neuropathic Symptoms and Signs (S-LANSS) Questionnaire

Aims to identify the pain of predominantly neuropathic origin, distinct from nociceptive pain, without the need for clinical examination. The S-LANSS is a 5-item questionnaire related to symptoms of pain and with two items related to clinical signs involving self-administered sensory tests for the presence of allodynia and decreased pin-prick sensation. The scores range from 0 to 24, with a score of 12 or greater suggesting neuropathic pain. ^10^

The validity and reliability of the Turkish version of these scales were developed.

### Data analysis

All statistical analyses were performed using the IBM SPSS Statistics (Version 20.0. Armonk, NY: IBM Corp.) software package program. Continuous variables were expressed as mean ± SD, and Student’s *t*-tests were used to compare the means between the two groups. For categorical variables, a Chi-Square test was used to test the differences between groups. Spearman’s correlation analysis was used for the correlation between neuropathy tests and disease characteristics. Unless otherwise stated, p < 0.05 was set as the level of significance.

## Results

The study included 51 patients (36 females, 15 males) and 47 controls (27 females, 20 males), and the two groups had similar sociodemographic characteristics (p < 0.05). In the patient group, 18 were on omalizumab and 18 were on antihistamines, while the remainder were not receiving treatment at the time of admission (Table 1).

**Table 1:** General demographic data and clinical features of the patient group.

	Patients (n = 51)	Controls (n = 47)	p
**Gender (Ratio of Female/Male)**	36/15	27/20	0.208
**Age (median 25–75pr)**	41 (29‒53)	39 (31‒53)	0.862
**BMI (median 25–75pr)**	26.72 (23.50‒31.25)	26.12 (23.23‒30.81)	0.644
**Education status, n (%)**			0.506
Primary school	18 (35.3%)	11(23.4%)	
Secondary school	4 (7.8%)	4 (8.5%)	
High school	10 (19.6%)	8 (17%)	
University	19 (37.3%)	24 (50%)	
**Additional disease, n (%)**			0.982
Hypertension	5 (9.8%)	4 (8.5%)	
Diabetes *mellitus*	6 (11.8%)	5 (10.6%)	
Guatr	8 (15.7%)	6 (12.8%)	
Others	3 (5.9%)	2 (4.3%)	
**Disease duration (years)**	3 (1‒9)	‒	‒
**Urticaria activity score (median 25–75pr)**	28 (14‒28)	‒	‒
**Finding of angioedema (n)**	20 patients	‒	‒
**Therapy of urticeria**			
None	15 (29.4%)	‒	‒
Antihistaminic	18 (35.3%)		
Omalizumab	18 (35.3%)		

BMI, Body Mass Index; p < 0.05 statistically significant.

The findings were given as median and interquartile range (25p‒75p).

An assessment of the results of the short-form McGill Pain Questionnaire revealed the scores in the sensory and affective domains, VAS scores and pain indices to be significantly higher in the patient group (p < 0.05 for all cases), while the overall pain assessment and sensory assessment based on the S-LANSS pain scale were also significantly higher in the patient group. Based on the assumption that scores of > 12 indicated neuropathy, 27 (53%) of the patients in the patient group and 8 (17%) in the control group were found to have neuropathy (p < 0.05). An analysis of the results of the questionnaire revealed the median total neuropathy score in the patient group to be 14, which was statistically compatible with the neuropathy findings (range: 0–33) (Table 2).

**Table 2:** Evaluation of McGill pain scale short form questions and S-LANSS pain questionnaires in groups.

	Patients (n = 51)	Controls (n = 47)	p
**McGill pain questionnaires**			
**Sensory score**	7 (3.75‒16.5)	0 (0‒3)	0.000
**Perceptual score**	2 (0‒6)	0 (0‒1)	0.000
**Index of pain**	11 (6‒14.25)	0 (0‒4)	0.000
**VAS**	0 (0‒6)	0 (0‒3)	0.001
**S-LANSS pain questionnaires**			
**Pain questionnaires**	11 (6‒14.25)	0 (0‒4)	0.000
**Sensory evaluation**	3 (0‒8)	0 (0‒0)	0.001
**Total score**	14 (6‒22)	0 (0‒5)	0.000
**Neuropathy accompanying person, n (%)**	27 (53%)	8 (17%)	0.000

VAS, Visual Analog Scale. The findings were given as median and interquartile range (25p‒75p).

An analysis of the correlations between the disease activation findings (duration of disease, frequency of attacks, urticaria activity score, and presence of angioedema) and neuropathy revealed a statistically strong positive correlation, especially with the urticaria activity score (p < 0.001, rho range: 0.524–0.969) (Table 3).

**Table 3:** Correlation between disease duration, attack frequency, urticaria activity score, finding of angioedema and neuropathy tests.

	Sensory score	Perceptual score	Index of pain	VAS	Pain questionnaires	Sensory evaluation	Total score
	p/rho	p/rho	p/rho	p/rho	p/rho	p/rho	p/rho
**Disease duration**	0.335	0.174	0.656‒0.065	0.464	0.656‒0.065	0.798‒0.037	0.738‒0.048
	0.139	0.195		0.105			
**Attack frequency**	0.510	0.596	0.300	0.990‒0.002	0.300	0.842	0.468
	0.095	0.077	0.150		0.150	0.029	0.105
**Urticaria activity score**	**0.000**	**0.000**	**0.000**	**0.000**	**0.000**	**0.000**	**0.000**
	0.572	0.524	0.969	0.533	0.969	0.894	0.523
**Finding of angioedema**	0.293	0.307	0.097	0.992	0.097	0.293	0.222
	0.152	0.147	0.238	0.001	0.238	0.152	0.121

Spearman's correlation analysis.

**Figure 1:**
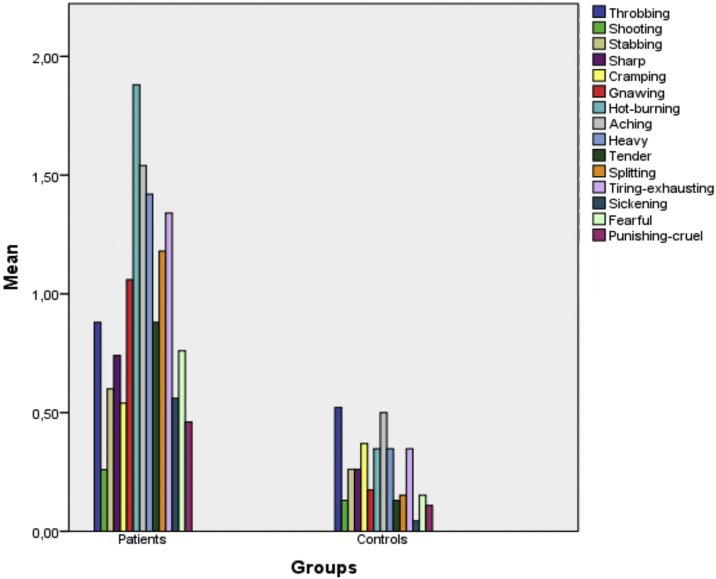
Bar graphs of the findings according to Mcgill Pain Questionnaires in the groups.

Fig. 1 presents a bar chart of the patient and control group symptoms according to the McGill Questionnaire items. As can be seen from the chart, the descriptors that were statistically strongly significant were aching, heavy, hurtful, gnawing and hot burning (p < 0.001 for all). As for the affective domain, the strongest significant descriptors were tiring-exhausting (p < 0.001), sickening and fearful (p = 0.001), while others were throbbing (p = 0.195), shooting (p = 0.591), stabbing (p = 0.041), sharp (p = 0.042), cramping (p = 0.591), splitting (p = 0.090) and punishing-cruel (p = 0.018).

## Discussion

In the present study significantly, higher scores were identified in the sensory and affective domains of the short-form McGill Pain Questionnaire, as well as the VAS scores and pain indices in the patient group. The overall pain assessments and sensory assessments identified from the S-LANSS pain scale were also significantly higher in the patient group. Neuropathy was detected in 27 and eight of the patient and control groups, respectively. There was a statistically strong positive correlation between the urticaria activity score, and the neuropathy test scores.

Neuropathic pain is a common problem in clinical practice that affects many aspects of daily life, and that is associated with poor overall health, reduced quality of life, poor sleep, and higher levels of anxiety and depression. The quality-of-life measurements of those with chronic neuropathic pain were found to be as low as in those with depression, coronary artery disease, recent myocardial infarction or uncontrolled diabetes mellitus.^11^

Mast cells are immunological cells that are distributed throughout different parts of the body with reported roles in several pathological conditions, such as hypersensitivity, atherosclerosis, pulmonary hypertension and infertility. There have also been studies reporting the importance of their roles in the pathogenesis of neuropathic pain of various etiologies^7^ Olsson^12^ first reported the role of mast cells in neuropathic pain and demonstrated the degranulation of mast cells in epineurium, perineurium and endoneurial connective tissue in response to trauma. Subsequent studies have revealed the role of increased mast cells in the regions surrounding the nerves and their mediators (primarily histamine and 5-HT) in the etiology of neuropathic pain.^13^, ^14^, ^15^ Considering the pathogenesis of CSU, it seems likely to be mainly a mast cell-derived disease. Cytokines such as histamine and platelet-activating factor, tryptase and leukotrienes released from mast cells cause sensory nerve activation, vasodilation and plasma extravasation.^16^ As can be seen, mast cells appear in the etiopathogenesis of diseases such as CSU and neuropathic pain. It was hypothesized in the present study that neuropathic pain-related symptoms may be more common in patients with CSU. To the best of our knowledge, however, there has been no study addressing this subject in literature to date. Consequently, the present study is the first to evaluate the relationship between CSU and symptoms of neuropathy.

It has been well established that sensitized mast cells can cause allergies and anaphylaxis, and mast cells are also known to contribute to several biological and pathological processes, such as vascular physiology, pain, itching and cancer.^17^, ^18^, ^19^ Among the previous studies in the literature evaluating the relationship between dermatological diseases and symptoms of neuropathy, Di Carlo et al. examined 118 psoriatic arthritis patients and applied questionnaires through which they detected symptoms of neuropathy in 25.4% of the patients.^20^ Garcovich et al. evaluated the neuropathic pain symptoms of 110 patients with hidradenitis suppurativa using questionnaires, and classified 33 (30%) of the 110 patients as having “likely neuropathic pain”, 32 (29.1%) with “possible neuropathic pain” and 45 (40.9%) with “unlikely neuropathic pain”. The pain and itching scores were found to be correlated, and the patients reported that their daily activities were significantly impaired due to pain. The authors emphasized the complex and multifactorial factors determining the pain phenotype in hidradenitis suppurativa.^21^ Eosinophilic perineural inflammation and abnormal mast cell innervation were demonstrated in the histopathology of the lesional and perilesional skin of patients with hidradenitis suppurativa, suggesting a potential anatomical correlate of “neuropathic” pain.^22^ Lipoxygenase-derived inflammatory mediators, eicosanoids and leukotriene B4 are increased in lesional skin, and are known to be inducers of peripheral inflammation and hyperalgesia.^23^ In light of this information, it is no surprise that patients with hidradenitis suppurativa tend to have accompanying symptoms of neuropathic pain.

Atopic dermatitis presents with severe itching, similar to CSU, and recent studies have reported accompanying pain symptoms in addition to itching in patients with atopic dermatitis, identifying a need to question patients in this regard.^24^, ^25^, ^26^ Pain was commonly described as “burning” and “stinging” by patients with atopic dermatitis^26^ who described their experience of itching as painful, throbbing, biting, stinging, burning, sharp, tingling, pinprick-like, and/or associated with crawling sensations.^24^, ^25^ All these clinical characteristics support the partial (but not fully) neuropathic component of itching.

Wallis et al. examined 35 patients with pachyonychia congenital – a rare autosomal dominant disorder – and assessed the severity and type of pain, as the main symptom in these patients, through questionnaires and quantitative sensory tests. The authors reported that 62% of the patients had neuropathic pain and the rest had nociceptive pain symptoms.^27^ Similarly, Brill et al. made a recent study of the origin of pain in patients with pachyonychia congenital in which 62 patients and 45 controls applied the McGill and Douleur Neuropathique-4 questionnaires and underwent sensory tests. The authors found that pain may be of neuropathic origin and suggested that patients could benefit from neuropathy-oriented treatments.^28^

This study has some limitations, the main ones being its cross-sectional study design, the small patient sample and the use of self-reported scales. Comorbidities such as type 2 diabetes could be associated with neuropathic pain, but these were present in only a small proportion of the sample, and there was no difference in the comorbidities of the two groups. Furthermore, no electrophysiological screening tests could be performed, as a further limitation. The questionnaires applied can be considered valid and reliable for use in screening tests, and in some cases, they may help identify disorders that cannot be detected by electrophysiology.

## Conclusion

In addition to itching, patients with CSU should be aware of the potential for accompanying neuropathic pain. In this chronic disease that is known to affect the quality of life, using an integrated approach with the patients and identifying accompanying problems are as important as treating the dermatological disorder. There is a need for prospective studies of this issue in which descriptive and methodological methods are applied together in large patient series to support the findings of the present study.

## Financial support

None declared.

## Authors’ contributions

Gülhan Gürel - Designed the study; recruited the participants; participated in the data collection; interpreted the data; drafted the first manuscript and all authors critically reviewed the paper.

Hikmet Saçmacı - Designed the study; participated in the data collection; did the statistical analysis; interpreted the data.

## Conflicts of interest

None declared.
